# Variable gene transcription underlies phenotypic convergence of hypoxia tolerance in sculpins

**DOI:** 10.1186/s12862-018-1275-1

**Published:** 2018-11-03

**Authors:** Milica Mandic, Marina L. Ramon, Aleeza C. Gerstein, Andrew Y. Gracey, Jeffrey G. Richards

**Affiliations:** 1 Department of Zoology, University of British Columbia, 6270 University Blvd, Vancouver, BC V6T 1Z4 Canada; 2 Department of Biological Sciences, University of Southern California, 3616 Trousdale Parkway, Los Angeles, CA 90089-0371 USA; 3 Department of Genetics, Cell Biology and Development, University of Minnesota, 321 Church Street, Minneapolis, MN 55455 USA; 4 Bamfield Marine Sciences Centre, 100 Pachena Dr, Bamfield, BC V0R 1B0 Canada

**Keywords:** Convergent evolution, Transcriptomics, Hypoxia, Fish, Sculpin

## Abstract

**Background:**

The degree by which mechanisms underlying phenotypic convergence are similar among taxa depends on the number of evolutionary paths available for selection to act upon. Likelihood of convergence will be influenced by an interplay of factors such as genetic architecture, phylogenetic history and population demography. To determine if there is convergence or divergence in mechanisms underlying phenotypic similarity, we assessed whether gene transcription patterns differed among species with similar levels of hypoxia tolerance.

**Results:**

Three species of marine fish from the superfamily Cottoidea (smoothhead sculpin [*Artedius lateralis*], sailfin sculpin [*Nautichthys oculofasciatus*] and Pacific staghorn sculpin [*Leptocottus armatus*]), all of which have previously been shown to share the same level of hypoxia tolerance, were exposed to short-(8 h) and longer-term (72 h) hypoxia and mRNA transcripts were assessed using a custom microarray. We examined hypoxia-induced transcription patterns in metabolic and protein production pathways and found that a high proportion of genes associated with these biological processes showed significant differences among the species. Specifically, the data suggest that the smoothhead sculpin, unlike the sailfin sculpin and the Pacific staghorn sculpin, relied on amino acid degradation rather than glycolysis or fatty acid oxidation to generate ATP during hypoxia exposure. There was also variation across the species in the transcription of genes involved in protein production (e.g. mRNA processing and protein translation), such that it increased in the smoothhead sculpin, decreased in the sailfin sculpin and was variable in the Pacific staghorn sculpin.

**Conclusions:**

Changes in metabolic and protein production pathways are part of the key responses of fishes to exposures to environmental hypoxia. Yet, species with similar overall hypoxia tolerance exhibited different transcriptional responses in these pathways, indicating flexibility and complexity of interactions in the evolution of the mechanisms underlying the hypoxia tolerance phenotype. The variation in the hypoxia-induced transcription of genes across species with similar hypoxia tolerance suggests that similar whole-animal phenotypes can emerge from divergent evolutionary paths that may affect metabolically important functions.

**Electronic supplementary material:**

The online version of this article (10.1186/s12862-018-1275-1) contains supplementary material, which is available to authorized users.

## Background

Natural selection has long been implicated as the evolutionary cause of convergent evolution of phenotypic traits. In theory, phenotypic convergence among species may evolve through similar or different genetic changes depending on the number of evolutionary paths available for selection to act upon. For example, genetic constraints, such as pleiotropy or limited available genetic variation may decrease the number of ways in which an animal can evolve in response to an environmental stressor, leading to similar genetic solutions [1–3]. In contrast, if genetic constraints are minimal and genetic variation is high, there may be a greater number of pathways available for evolution, and different genetic solutions could underlie convergent phenotypes. There has been increased effort in recent years to identify the underlying genetic and molecular mechanisms that yield trait convergence, with the long-term goal of understanding the principles that govern the genetic basis of phenotypic convergence [4–8].

Genetic convergence has been examined across hierarchical levels in a number of different animal systems [4, 9, 10], revealing the complexity of how similar phenotypes can evolve in response to the same ecological pressure. Parallel genotypic adaptation can involve differing levels of biological organization: from the exact same mutation in the same gene, to different mutations in the same gene, through different genes in the same gene network or pathway [11, 12]. With the advent of high-throughput transcriptomics, similarities in gene expression in pathways and networks across populations or species adapting to similar environmental stressors have been identified [5, 13–18]. Focusing on transcriptomics is important because it provides an understanding of the variation in genome regulation in response to the environment, ultimately providing an important link in elucidating genotype-environment interactions, phenotypic plasticity and phenotypic evolution [19–21].

Environmental hypoxia (i.e., low O_2_ levels) is well known to have significant ecological and physiological consequences in animals, particularly in aquatic organisms, which can experience hypoxia frequently due to the low solubility of O_2_ in water. As a result, there have been multiple, independent origins of hypoxia tolerance among diverse fish lineages [22]. Accumulating evidence suggest that hypoxic survival in fish is linked to the maintenance of energy supply and demand [23], which is most often achieved through a combination of modifications for O_2_ uptake to sustain aerobic metabolism, increased reliance on O_2_-independent ATP production (e.g. glycolysis), as well as decreased energy demand through a coordinated suppression of energetically expensive processes such as protein production [24–26]. Modifications to these processes can be brought about through differences in gene transcription [27, 28], post-translational modifications [29], as well as physiological [30, 31] and behavioural [32] changes. Although a great deal is known about some of the mechanisms that confer hypoxia tolerance, no study has directly examined whether species with similar levels of hypoxia tolerance show convergence in the underlying mechanisms. Specifically, have species in the wild that are subjected to the same degree of hypoxia evolved these complex phenotypes via convergence at the level of gene transcription in the key hypoxic response pathways?

We exposed three species of marine fish from different clades in the superfamily Cottoidae [33] to hypoxia for up to 72 h and sampled liver for transcriptomics analysis. The smoothhead sculpin (*Artedius lateralis*), sailfin sculpin (*Nautichthys oculofasciatus*) and Pacific staghorn sculpin (*Leptocottus armatus*) all inhabit the high subtidal environment, which is susceptible to periodic decreases in O_2_, and show the same level of whole-animal hypoxia as indicated by the critical O_2_ tension of O_2_ consumption rate (P_crit_), a complex phenotype [34]. Available data on the smoothhead sculpin and the Pacific Staghorn sculpin shows that the species also have similar time to loss of equilibrium when exposed to low O_2_ levels enforcing the idea that these species have the same level of tolerance to environmental hypoxia [35].

The hypoxia tolerances of the focal species are intermediate between hypoxia tolerant species that live in the tidepool environment and experience large and frequent O_2_ fluctuations and the hypoxia intolerant species that live in the deeper, O_2_ stable environments. Using the hypoxia tolerant tidepool sculpin (*Oligocottus maculosus*) and hypoxia intolerant silverspotted sculpin (*Blepsias cirrhosis*), we have previously shown that these species have highly divergent patterns of gene transcription in both the induction or repression patterns of the transcriptional response and the timing of the hypoxia-induced transcriptional change [36]. It remains unknown, however, whether species with similar levels of hypoxia tolerance also show divergence in gene transcription patterns in pathways known to impact hypoxic survival, which would suggest that differences in genome regulation can underlie equivalent complex phenotypes such as hypoxia tolerance.

## Methods

### Experimental animals

Individuals of *Artedius lateralis* (smoothhead sculpin) and *Leptocottus armatus* (Pacific staghorn sculpin) were collected in July and August near the Bamfield Marine Sciences Centre (BMSC), Bamfield, British Columbia. Smoothhead sculpins and Pacific Staghorn sculpins were captured at Ross Islets (48°52′24.0”N 125°09′42.0”W) and Bamfield Inlet (48°49′06.1”N 125°08′30.9”W) using seine nets during low tide and baited minnow traps. The two collection sites are prone to decreases in O_2_ as a result of the ebb and flow of the tides and both sculpin species have been found in the locations during low tide cycle, indicating that the species are exposed to similar periodic environmental hypoxia (personal observations). Sculpins collected at BMSC were transported to The University of British Columbia and a third species, *Nautichthys oculofasciatus* (sailfin sculpin) was acquired from the Vancouver Aquarium, British Columbia, Canada. The sailfin sculpins were first generation offspring from wild fish caught in the waters around Stanley Park, Vancouver, BC. All three species were held in fully aerated recirculating seawater at 12 **°**C and 30 ppt salinity for at least 2 months prior to experimentation. Fish were fed daily with prawns and krill up to 24 h before the experiments when feeding ceased. Animal collections were approved by Department of Fisheries and Oceans (License XR 143 2010) and Bamfield Marine Sciences Centre animal care committee (AUP RS-11-17) and followed the appropriate guidelines. All experiments were approved by The University of British Columbia animal care committee (A09–0611).

### Critical O_2_ tension

Critical O_2_ tension (P_crit_; water *P*O_2_ at which an animal no longer maintains routine O_2_ consumption rate and the rate decreases with a decline in water *P*O_2_) is related to hypoxia tolerance in sculpins [35] and P_crit_ values for smoothhead and Pacific staghorn sculpins taken from [34] were 35.7 ± 6.9 Torr and 37.4 ± 1.2 Torr, respectively. Following protocols described in detail in [37], we determined that the P_crit_ for sailfin sculpin was 35.5 ± 2.3 Torr (*n* = 6) at 12 **°**C and 30 ppt salinity. The P_crit_ values were not statistically different between species (ANOVA, *p* = 0.900).

### Hypoxia exposure

For each species, individuals were transferred into four separate, 136 l tanks of a custom built recirculating system, the Low Oxygen Control and Monitoring Aquatic Research System (Integrated Aqua Systems, Inc.). Fish were allowed to recover in air-saturated water for 24 h, after which O_2_ levels were decreased to 23 Torr, a level of hypoxia corresponding to 65% P_crit_ of all three species. Liver was sampled from each fish at 3, 8, 24, 48 and 72 h of hypoxia and two normoxic fish were also sampled at each of the hypoxic time points. Fish were removed from the tank and immediately euthanized by exposure to an overdose of benzocaine (125 mg/mL; Sigma-Aldrich). Tissue was dissected, flash frozen in liquid N_2_ and stored at − 80 **°**C until analysis.

### Microarray experiments

The protocols used for RNA extraction, reverse-transcription of RNA to cDNA, fluorescent labeling of the cDNA and hybridization of the microarrays are described in detail in [36]. Briefly, RNA was extracted from the liver of each species using TRIzol (Invitrogen). Total RNA was then purified using glass-fibre filter columns (Qiagen) and reverse transcribed using MMLV-reverse transcriptase (Epicentre). The cDNA samples were purified using QIAquick PCR purification kits (Qiagen) and each sample was labeled separately with Cy5 and Cy3 dyes. Fluorescently labeled cDNA samples were hybridized to a custom cDNA array from woolly sculpin (*Clinocottus analis*). A total of 25 samples per species were used: 5 individuals sampled at normoxia and 4 individuals sampled at each hypoxic time-point. Hybridizations were only performed between 2 samples from the same species hybridized on the woolly sculpin array. We used an interwoven loop design [38], where each sample was hybridized to either 2 or 4 arrays with fluor-reversal, creating a separate loop for each species (Additional file 1: Figure S1).

### Microarray and statistical analysis

The custom woolly sculpin array is comprised of approximately 9600 cDNA clones (array elements; [36]). A total of 1700 clones were sequenced and the resulting non-redundant contigs were annotated with the gene name only if the matched sequences yielded a hit with an E-value of <1e-^4^ in SwissProt (for details see [36]). For the purpose of this manuscript, the term ‘clone’ refers to all array elements (sequenced and non-sequenced), while the term ‘gene’ refers only to the sequenced and annotated clones. Sequence divergence between the woolly sculpin used to create the microarray platform and the sculpin species under investigation may result in poor hybridization at certain clones; however, we attempted to minimize the effect of poor hybridization by only considering clones with expression levels > 2 standard deviations above background for any species. Using this criterion, 3294 clones of the possible 9600 clones were analyzed.

Raw data were log_2_-transformed and lowess normalized using the MAANOVA package [39] in the R programming language [40]. To test for the effects of time in hypoxia, differential expression for each clone across time-points was detected using linear mixed models (‘array’ as a random effect and ‘dye’ and ‘time’ as fixed effects) with 100 permutations and the F-test statistic. False discovery rate [41] in the q value package [42] in R was used to determine significance level (set at *q* < 0.01). Clones showing a main effect of time in hypoxia in at least one of the species of sculpin (2734 clones in total; 1670 clones for smoothhead sculpin, 1183 clones for sailfin sculpin and 1778 clones for Pacific staghorn sculpin) were first categorized into annotated and non-annotated groups. Annotated genes were assigned to gene ontology biological process categories using searches in EMBL-EBI QuickGO (www.ebi.ac.uk/QuickGo/) and Uniprot (http://www.uniprot.org). Only genes associated with energy metabolism and protein production were further analyzed to determine if the transcription patterns differed among the species.

**Fig. 1:**
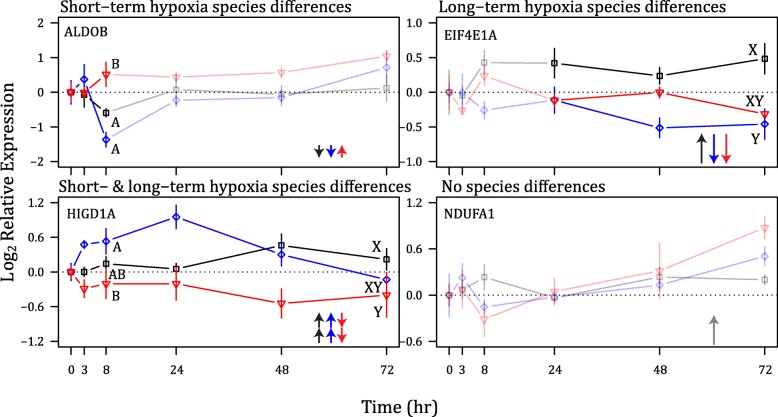
Transcript levels of a representative gene for each category of the transcription response. Smoothhead sculpin (black line, square symbol), sailfin sculpin (blue line, diamond symbol) and Pacific staghorn sculpin (red line, inverted triangle symbol) were exposed to 72 h of hypoxia. Opaque lines represent the portion of the time-course that is different among the species, while transparent lines represent the portion of the time-course for which transcription does not differ among the species. Letters represent significant difference in transcript levels between species (A-B represent difference in the short term hypoxia and X-Y represent differences in the long term hypoxia). Full gene names are as follows: fructose-bisphosphate aldolase B (ALDOB), eukaryotic translation initiation factor 4E-1A (EIF4E1A), HIG1 domain family member 1A (HIGD1A) and NADH dehydrogenase 1 alpha subcomplex subunit 1 (NDUFA1)

To determine whether the direction and/or rate of transcriptional response varied among species we compared the slope of the transcription responses for short-term hypoxia exposure (0, 3 h, 8 h) and long-term hypoxia exposure (0, 24 h, 48 h, 72 h). The time-course was partitioned into short- and long-term responses because: 1) daily tidal events are the primary cause of hypoxic exposure in the environments inhabited by these species, resulting in ecologically relevant hypoxic exposure of up to 8 h; 2) shallow coastal environments are increasingly influenced by anthropogenic eutrophication resulting in longer-term, physiologically challenging hypoxia exposures [43]; and 3) our previous work comparing the responses of hypoxia-tolerant and -intolerant sculpins demonstrated a strong effect of time on transcription with differences between the early and the late hypoxia exposure [36]. A maximum-likelihood model was applied to the data to determine the best fit intercept and linear slope for each species over short- and long-term hypoxia, using the subplex method of the optim function, as implemented in the find.mle routine of the diversitree package [44]. For each species pair (smoothhead sculpin vs. sailfin sculpin, smoothhead sculpin vs. Pacific staghorn sculpin, and sailfin sculpin vs. Pacific staghorn sculpin), we then used a likelihood-ratio test to determine whether the slopes were significantly different by comparing the full likelihood model (which allowed both species to have different slopes) to a constrained model with a single slope. If the drop in log-likelihood between the full and the constrained model was > 1.92 (the critical value for a chi-square distribution with 1 degree of freedom and an alpha level of 0.05) we then rejected the hypothesis that the slopes were the same for the two species. From this analysis, we broadly classified the data into 4 categories of transcriptional response (Fig. 1): differences among the species during short-term hypoxia exposure, differences among the species during long-term hypoxia exposure, differences among the species in both the short- and long-term hypoxia exposure, and no differences among the species.

### Wild caught versus lab-reared species

In this study, individuals of the smoothhead sculpin and the Pacific staghorn sculpin were wild-caught, while individuals of the sailfin sculpin were first generation lab-reared. In theory this may complicate interpretation, as the wild-caught species may have been exposed to hypoxia during development, which can have consequences on the adult hypoxic phenotype [45]. However, an examination of the patterns of all transcriptionally-responsive clones using heat maps across the three species produced no clear indication to suggest that the overall transcriptional response of the sailfin sculpin differed to a great degree from the other two species (data not shown). Of course, this does not exclude the possibility that some of the patterns described in this study are a result of differences in developmental plasticity. One could potentially get around the effects of developmental plasticity by examining first generation lab-reared individuals for all species (caveat: wild-caught individuals of many sculpin species are difficult to breed under laboratory conditions), although epigenetic effects can persist across many generations increasing the difficulty of unraveling the genetic mechanisms of survival in hypoxia.

## Results and discussion

To determine whether hypoxia tolerance is a result of convergence of traits at the underlying molecular level we examined temporal patterns of gene transcription in response to hypoxia exposure in three species of sculpin from superfamily Cottoidae. Intriguingly, in spite of their similar overall hypoxia tolerance, genes associated with energy metabolism and protein production, the key pathways involved in hypoxia survival, have divergent transcription patterns (59% and 73% of genes respectively) among the three species. This indicates that different genomic solutions were used to achieve the same phenotypic outcome.

### Energy metabolism

**Fig. 2:**
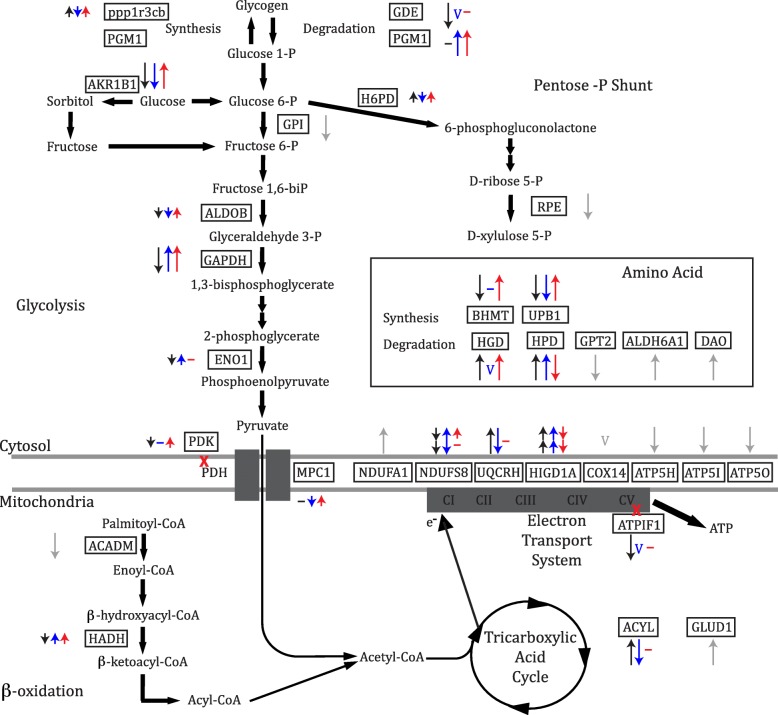
Schematic representation of genes associated with metabolism. Arrows represent gene transcription changes in response to hypoxia in the smoothhead sculpin (black), sailfin sculpin (blue) and Pacific staghorn sculpin (red). Gray arrows represent genes with similar transcription patterns among the species. Short arrows indicate transcription patterns during short-term hypoxia, long arrows indicate transcription patterns during long-term hypoxia, double arrows indicate transcription patterns during short- and long-term hypoxia, a horizontal line indicates no change from normoxia and the letter ‘v’ represents a variable response. Full names for each gene abbreviation as well as complete transcription profiles of the genes are found in Additional files 2, 4 and 5 (Figures S2, S4-S5).

Transcription of metabolic genes varied among the three sculpin species, suggesting that the species may rely on different metabolic pathways to generate ATP during hypoxia exposure. These transcriptional data strongly indicate that the three sculpin species use different and diverse metabolic pathways for ATP generation during hypoxia exposure (Fig. 2), challenging the classic dogma that hypoxia survival is solely dependent on the Pasteur effect and the up-regulation of glycolytic function for ATP production [22].

In the smoothhead sculpin, transcript level changes in response to hypoxia suggest this species relies on enhanced amino acid degradation rather than glycolysis or fatty acid oxidation to generate substrates for the tricarboxylic acid (TCA), and thereby support ATP generation by oxidative phosphorylation (Fig. 2). Transcript levels of genes associated with amino acid degradation and the TCA cycle showed an increase in transcript levels during long-term hypoxia, while transcript levels of genes associated with amino acid synthesis decreased as compared to normoxic levels (Fig. 2). A reduced reliance on glycolysis during hypoxia is also consistent with the observation of a reduction in the transcript levels of glycolytic genes (e.g., fructose-bisphosphate aldolase B [ALDOB], glyceraldehyde-3-phosphate dehydrogenase [GAPDH]) and genes associated with glycogen degradation (glycogen debranching enzyme [GDE]; Fig. 2, Additional file 2: Figure S2). The smoothhead sculpin also had a reduction in transcript levels for genes associated with fatty acid oxidation during short- and long-term hypoxia exposure (e.g., hydroxyacyl-coenzyme A dehydrogenase [HADH]; Fig. 2, Additional file 2: Figure S2).

If gene transcript levels relate to biological function, these data suggest that in addition to the typical hypoxia-induced reliance on glycolysis for ATP generation, prolonged hypoxia exposure in smoothhead sculpins induces a greater reliance on amino acid catabolism as a source of ATP. An increased reliance on O_2_-dependent ATP production via amino acid catabolism may seem like a surprising response for an organism to have in an O_2_ deprived environment, but previous studies have shown enhanced amino acid catabolism during hypoxia in at least two other fish species (*Tilapia mossambica*; [46] and *Oncorhynchus mykiss*; [47]).

In contrast to the smoothhead sculpin, both the sailfin sculpin and the Pacific staghorn sculpin showed a general increase in the transcription of glycolytic genes during short- and long-term hypoxia exposure (e.g., GAPDH; Fig. 2, Additional file 2: Figure S2). This would suggest an increased reliance on glycolysis during hypoxic exposure, a response that has been well documented in fishes, reptiles and mammals [48–51]. Both species also showed a short-term increase in transcript levels of genes associated with fatty acid oxidation (e.g., HADH) followed by a decrease in transcript levels during long-term hypoxia (e.g., medium-chain specific acyl-CoA dehydrogenase [ACADM]; Fig. 2, Additional file 2: Figure S2). The increase or lack of change in transcription of fatty acid oxidation genes during short-term hypoxia exposure followed by a decrease in the transcription of the genes during long-term hypoxia exposure have been noted in the heart of rats [52]. The decline in fatty acid oxidation during hypoxia has been thought to contribute to a decrease in energy expenditure as well as an avoidance of lipotoxicity [53, 54].

The sailfin and Pacific staghorn sculpins showed divergent responses in gene transcription associated with other metabolic processes. Transcription patterns of genes associated with amino acid synthesis and degradation in the sailfin sculpin were similar to those of the smoothhead sculpin, with a decrease in beta-ureidopropionase [UPB1] and an increase in 4-hydroxyphenylpyruvate dioxygenase [HPD] (Fig. 2, Additional file 2: Figure S2). However, this was in contrast to the Pacific staghorn sculpin, which showed transcriptional patterns that suggest both increased amino acid synthesis and degradation (Fig. 2). We also identified a significant increase in genes involved in glycogen degradation in the sailfin sculpin while in the Pacific staghorn sculpin there was shift from glycogen synthesis in the short-term to glycogen degradation during long-term hypoxia exposure.

### Protein production, protein localization and protein folding

**Fig. 3:**
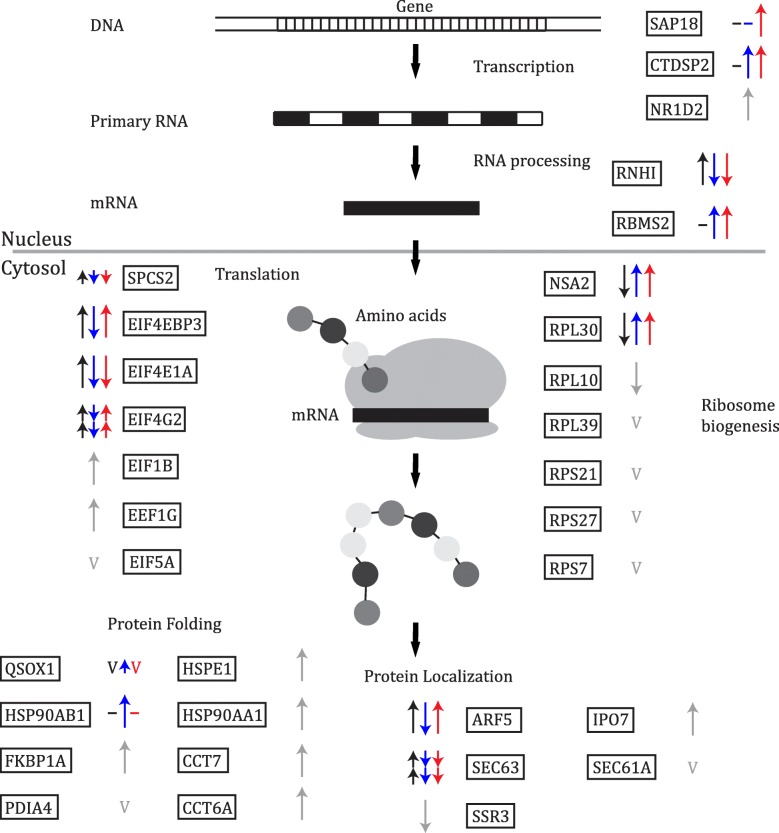
Schematic representation of genes associated with protein production, localization and folding. Arrows represent gene transcription changes in response to hypoxia in the smoothhead sculpin (black), sailfin sculpin (blue) and Pacific staghorn sculpin (red). Gray arrows represent genes with similar transcription patterns among the species. Short arrows indicate transcription patterns during short-term hypoxia, long arrows indicate transcription patterns during long-term hypoxia, double arrows indicate transcription patterns during short- and long-term hypoxia, a horizontal line indicates no change from normoxia and the letter ‘v’ represents a variable response. See Additional files 3, 4 and 5 (Figures S3-S5) for full gene names and complete transcription profiles of the genes

Protein production is a significant energy sink and inhibition of this process during hypoxia is thought to be an important step in conserving the limited energy stores [24–26]. Multiple steps are involved in cellular protein production, including gene transcription, RNA processing and translation (Fig. 3). Differences in the transcriptional patterns of genes associated with these processes suggest that during short and long-term hypoxia exposure the smoothhead sculpin increased protein production through up-regulation of genes associated with translation, while the sailfin sculpin showed a corresponding decrease in these same genes (e.g., eukaryotic translation initiation factor 4 gamma 2 [EIF4G2] and eukaryotic translation initiation factor 4E-1A [EIF4E1A]; Fig. 3, Additional file 3: Figure S3). The Pacific staghorn had a variable response in genes associated with protein production, as highlighted by the transcriptional patterns of both the genes associated with RNA processing (e.g. ribonuclease inhibitor [RNHI] versus RNA-binding motif, single-stranded-interacting protein 2 [RBMS2]) and genes associated with translation (e.g. EIF4G2 versus EIF4E1A; Fig. 3, Additional file 3: Figure S3).

To fully examine protein production, it is also important to consider steps such as protein folding, targeting and localization. Although transcription patterns in a large proportion of genes associated with transcription, RNA processing and translation differed among the species, the majority of genes associated with protein localization and protein folding were transcriptionally similar among the species (Fig. 3, Additional file 4: Figure S4, Additional file 5: Figure S5). In particular, all three species showed an increase in transcription of genes associated with protein folding, especially during long-term hypoxia exposure. This is consistent with previous work that shows hypoxia exposure increases protein-folding chaperones in the liver of rats (heat shock protein 70; [55]) and in the Chinese shrimp (heat shock protein 90; *Fenneropenaeus chinensis*; [56]).

### Linking gene transcription to phenotype

From genotype to phenotype, variation in gene transcription is important to consider as it can be heritable and an outcome of both adaptive and neutral evolutionary processes [20, 57–59]. However, the relationship between gene transcription and phenotype is complex and causally linking variation in gene transcription to phenotypic variation can be difficult. Gene transcription is but one step of many along the pathway towards building a phenotype such as hypoxia tolerance. For example, hypoxia exposure has a significant impact on the translation efficiency of genes [60], and it is possible that the variation among the species at gene transcription level is accentuated or diminished at subsequent levels of biological organization. Therefore, convergence may be occurring not only at the level of hypoxia tolerance but in the underlying metabolic and protein production pathways among the three species of sculpin. While further work is required to determine if there is convergence at subsequent levels of biological organization, it is clear that there is divergent variation in gene transcription underlying hypoxia tolerance among the three species of sculpin.

### Comparisons with a hypoxia tolerant sculpin

Our previous work showed that the transcription patterns in the hypoxia-tolerant tidepool sculpin aligned with the characteristics that have long been attributed to hypoxia-tolerant animals, including an increase in transcript levels of glycolytic genes and a decrease in transcripts of genes associated with energetically expensive processes such as cell proliferation [36]. The three intermediate–tolerant species investigated in this study shared some of the same transcription patterns as the tidepool sculpin in genes considered important for hypoxia tolerance. For example, similar to the tidepool sculpin, the smoothhead sculpin showed a decrease in transcription of genes involved in some of the pathways that require O_2_, such as fatty acid oxidation, while the sailfin sculpin showed a decrease in protein production, and both the sailfin sculpin and Pacific staghorn sculpin showed an increase in glycolysis (Fig. 2, Fig. 3). Overall, there was significant variation among the three species in transcription patterns for genes that were a priori predicted to be important in hypoxia. The transcriptional patterns thus strongly suggest that the three species of sculpin have evolved similar levels of hypoxia tolerance via different underlying mechanisms.

### Short-term versus long-term responses

Time is an important factor to consider when examining changes in gene transcription [19]. In our previous work [36] we showed that time in hypoxia had a strong effect on gene transcription, and that hypoxia-tolerant and -intolerant sculpins showed different temporal patterns of gene transcript levels. The short-term (8 h) hypoxia exposure is an ecologically relevant time-frame for species inhabiting the intertidal, while the longer duration of hypoxia lasting for several days is a physiological challenge that tests the species’ capacities for hypoxic survival. Therefore, it is not surprising that different mechanisms may shape the hypoxic response between the short and long exposures to hypoxia and we predicted that in the current study differences in transcription patterns among the species would occur in both the short- and long-term hypoxia exposure. Indeed, 16% of genes showed differences among species during short-term hypoxia exposure, 33% during long-term hypoxia exposure and 6% showed transcriptional differences across both time scales in pathways associated with energy production and protein production (percentages are based on genes represented in the figures that are expected to be a priori involved in processes related to hypoxia). As an example, transcription of glycolytic genes differed among species in both short- and long-term hypoxia, although most genes differed only in one time frame or the other (Fig. 2). Divergent transcriptional responses as well as strong temporal effects thus influence the underlying mechanisms of hypoxia tolerance in sculpins. However, caution should be noted, as these species were exposed to a single bout of hypoxia whereas in the wild the species encounter hypoxia on a cyclical basis. This may have an impact on the transcriptional patterns and a critical future step is to examine the transcriptional response of metabolic and protein production genes over time in species exposed to tidal-influenced cyclical hypoxia.

## Conclusions

We found that three species of sculpin with similar hypoxia tolerance had very different patterns of transcription of genes involved in metabolism and protein production. These biological processes are known to have a significant influence on hypoxic survival and divergent transcription patterns underlying these traits suggest that different genetic changes were selected for during the evolution of intermediate hypoxia tolerance in the three species of sculpin. Our results indicate that there are multiple genomic pathways to achieve the same phenotype, and indicate that similarities in hypoxia tolerance among sculpins is due to phenotypic convergence rather than species relatedness.

## Additional files

Additional file 1: **Figure S1.** Hybridization loop of 3 species of sculpin. Samples are identified first by the hours in hypoxia followed by the number of the biological replicate. (PDF 294 kb)

Additional file 2: **Figure S2.** Transcript levels of genes associated with metabolism. Colours and symbols represent different species: the smoothhead sculpin (black line, square symbol), sailfin sculpin (blue line, diamond symbol) and Pacific staghorn sculpin (red line, inverted triangle symbol) exposed to 72 h of hypoxia. Solid lines represent the genes for which transcription significantly changed in response to hypoxia for a given species (*q* < 0.01) and dashed lines represent genes for which transcription did not significantly change in response to hypoxia. Opaque lines represent the portion of the time-course that is the focus of the difference among the species, while transparent lines represent the portion of the time-course for which transcription does not differ among the species. Letters represent significant difference in transcript levels between species (A-B represent difference in the short term hypoxia and X-Z represent differences in the long term hypoxia). Data are represented as mean ± SE. The genes in the panels are classified in the following categories: glycogen metabolic process (protein phosphatase 1 regulatory subunit 3C-B [ppp1r3cb], glycogen debranching enzyme [GDE] and phosphoglucomutase-1 [PGM1]), glycolysis (fructose-bisphosphate aldolase B [ALDOB], alpha-enolase [ENO1] and glyceraldehyde-3-phosphate dehydrogenase [GAPDH], polyol pathway (aldose reductase [AKR1B1]), the pentose-phosphate shunt (GDH/6PGL endoplasmic bifunctional protein [H6PD]), pyruvate metabolic process (pyruvate dehydrogenase kinase isozyme 4 [PDK] and mitochondrial pyruvate carrier 1 [MPC1]), fatty acid oxidation (hydroxyacyl-coenzyme A dehydrogenase [HADH]), tricarboxylic acid cycle (ATP-citrate synthase [ACYL]), oxidative phosphorylation (cytochrome b-c1 complex subunit 6 [UQCRH], ATPase inhibitor [ATPIF1], NADH dehydrogenase iron-sulfur protein 8 [NDUFS8] and HIG1 domain family member 1A [HIGD1A]), amino acid degradation (homogentisate 1,2-dioxygenase [HGD] and 4-hydroxyphenylpyruvate dioxygenase [HPD]) and amino acid biosynthesis (betaine—homocysteine S-methyltransferase 1 [BHMT] and beta-ureidopropionase [UPB1]). (PDF 458 kb)

Additional file 3: **Figure S3.** Transcript levels of genes associated with protein production, localization and folding. Colours and symbols represent different species: the smoothhead sculpin (black line, square symbol), sailfin sculpin (blue line, diamond symbol) and Pacific staghorn sculpin (red line, inverted triangle symbol) exposed to 72 h of hypoxia. The genes in the panels are classified in the following categories: transcriptional repression (histone deacetylase complex subunit SAP18 [SAP18] and carboxy-terminal domain RNA polymerase II polypeptide A small phosphatase 2 [CTDSP2]), RNA metabolic process (ribonuclease inhibitor [RNHI]), RNA processing (RNA-binding motif, single-stranded-interacting protein 2 [RBMS2] and ribosome biogenesis protein NSA2 homolog [NSA2] and 60S ribosomal protein L30 [RPL30]), translation (signal peptidase complex sunbunit 2 [SPCS2]), translational initiation (eukaryotic translation initiation factor 4E-binding protein 3 [EIF4EBP3], eukaryotic translation initiation factor 4E-1A [EIF4E1A] and eukaryotic translation initiation factor 4 gamma 2 [EIF4G2]), protein localization (ADP-ribosylation factor 5 [ARF5] and translocation protein SEC63 homolog [SEC63]), and protein folding (sulfhydryl oxidase 1 [QSOX1] and heat shock protein HSP 90-beta [HSP90AB1]). See **Figure S1** legend for more detail. (PDF 346 kb)

Additional file 4: **Figure S4.** Genes for which all three species showed similar significant changes in transcription in response to 72 h of hypoxia. The smoothhead sculpin is represented by the black line and square symbol, the sailfin sculpin is represented by the blue line and diamond symbol and the Pacific staghorn sculpin is represented by the red line and inverted triangle symbol. Data are represented as mean ± SE. The genes in the panels are classified in the following categories: pentose-phosphate shunt (ribulose-phosphate 3-epimerase [RPE]), fatty acid oxidation (medium-chain specific acyl-CoA dehydrogenase [ACADM]), oxidative phosphorylation (NADH dehydrogenase 1 alpha subcomplex subunit 1 [NDUFA1] and ATP synthase subunit d [ATP5H]), translational initiation (eukaryotic translation initiation factor 1b [EIF1B]), protein localization (importin-7 [IPO7] and translocon-associated protein subunit gamma [SSR3]) and protein folding (peptidyl-prolyl cis-trans isomerase FKBP1A [FKBP1A], protein disulfide-isomerase A4 [PDIA4] and T-complex protein 1 subunit zeta [CCT6A]). (PDF 136 kb)

Additional file 5: **Figure S5.** Similar transcription patterns in genes for which only a subset of species showed a significant effect of time in hypoxia. The smoothhead sculpin is represented by the black line and square symbol, the sailfin sculpin is represented by the blue line and diamond symbol and the Pacific staghorn sculpin is represented by the red line and inverted triangle symbol. Solid lines represent the genes for which transcription significantly changed in response to hypoxia for a given species (*q* < 0.01) and dashed lines represent genes for which transcription did not significantly change in response to hypoxia. Data are represented as mean ± SE. The genes in the panels are classified in the following categories: glycolysis (glucose-6-phosphate isomerase [GPI]), tricarboxylic acid cycle (glutamate dehydrogenase [GLUD1]), oxidative phosphorylation (cytochrome c oxidase assembly protein COX14 [COX14], ATP synthase subunit e [ATP5I] and ATP synthase subunit O [ATP5O]), amino acid degradation (alanine aminotransferase 2 [GPT2], methylmalonate-semialdehyde dehydrogenase, acylating [ALDH6A1] and D-amino-acid oxidase [DAO]), transcriptional repression (nuclear receptor subfamily 1 group D member 2 [NR1D2]), ribosomal biogenesis (60S ribosomal protein L10 [RPL10], 60S ribosomal protein L39 [RPL39], 40S ribosomal protein S21 [RPS21], 40S ribosomal protein S27 [RPS27] and 40S ribosomal protein S7 [RPS7]), translational elongation (elongation factor 1-gamma [EEF1G] and eukaryotic translation initiation factor 5A-1 [EIF5A]), protein folding (10 kDa heat shock protein [HSPE1], heat shock protein HSP 90-alpha [HSP90AA1] and T-complex protein 1 subunit eta [CCT7]) and protein localization (protein transport protein Sec61 subunit alpha [SEC61A]). (PDF 194 kb)

## Abbreviations

ACADM: Medium-chain specific acyl-CoA dehydrogenase
ACYL: ATP-citrate synthase
AKR1B1: Polyol pathway aldose reductase
ALDH6A1: Methylmalonate-semialdehyde dehydrogenase, acylating
ALDOB: Fructose-biphosphate aldolase B
ARF5: ADP-ribosylation factor 5
ATP: Adenosine triphosphate
ATP50: ATP synthase subunit O
ATP5H: ATP synthase subunit d
ATP5I: ATP synthase subunit e
ATPIF1: ATPase inhibitor
BHMT: Betaine—homocysteine S-methyltransferase 1
CCT6A: T-complex protein 1 subunit zeta
CCT7: T-complex protein 1 subunit eta
COX14: Cytochrome c oxidase assembly protein COX14
CTDSP2: Carboxy-terminal domain RNA polymerase II polypeptide A small phosphatase 2
DAO: D-amino-acid oxidase
EEF1G: Elongation factor 1-gamma
EIF1B: Eukaryotic translation initiation factor 1b
EIF4E1A: Eukaryotic translation initiation factor 4E-1A
EIF4EBP3: Eukaryotic translation initiation factor 4E-binding protein 3
EIF4G2: Eukaryotic translation initiation factor 4 gamma 2
EIF5A: Eukaryotic translation initiation factor 5A-1
ENO1: Alpha-enolase
FKBP1A: Peptidyl-prolyl cis-trans isomerase FKBP1A
GAPDH: Glyceraldehyde-3-phosphate dehydrogenase
GDE: Glycogen debranching enzyme
GLUD1: Glutamate dehydrogenase
GPI: Glucose-6-phosphate isomerase
GPT: Alanine aminotransferase 2
HADH: Hydroxyacyl-coenzyme A dehydrogenase
HGD: Homogentisate 1,2-dioxygenase
HIGD1A: HIG1 domain family member 1A
HPD: 4-hydroxyphenylpyruvate dioxygenase
HSP90AA1: Heat shock protein HSP 90-alpha
HSP90AB1: Heat shock protein HSP 90-beta
HSPE1–10 kDa: Heat shock protein
IPO7: Importin-7
MPC1: Mitochondrial pyruvate carrier 1
NDUFA1: NADH dehydrogenase 1 alpha subcomplex subunit 1
NDUFS8: NADH dehydrogenase iron-sulfur protein 8
NR1D2: Nuclear receptor subfamily 1 group D member 2
NSA2: Ribosome biogenesis protein NSA2 homolog
P_crit_: Critical O_2_ tension; water *P*O_2_ at which an animal no longer maintains routine O_2_ consumption rate and the rate decreases with a decline in water *P*O_2_
PDIA4: Protein disulfide-isomerase A4
PDK: Pyruvate dehydrogenase kinase isozyme 4
PGM1: Phosphoglucomutase-1
*P*O_2_: Partial pressure of oxygen
ppp1r3cb: Protein phosphatase 1 regulatory subunit 3C-B
QSOX1: Sulfhydryl oxidase 1
RBMS2: RNA-binding motif, single-stranded-interacting protein 2
RNHI: Ribonuclease inhibitor
RPE: Ribulose-phosphate 3-epimerase
RPL10: 60S ribosomal protein L10
RPL30: 60S ribosomal protein L30
RPL39: 60S ribosomal protein L39
RPS21: 40S ribosomal protein S21
RPS27: 40S ribosomal protein S27
RPS7: 40S ribosomal protein S7
SAP18: Histone deacetylase complex subunit SAP18
SEC61A: Protein transport protein Sec61 subunit alpha
SEC63: Translocation protein SEC63 homolog
SPCS2: Signal peptidase complex sunbunit 2
SSR3: Translocon-associated protein subunit gamma
TCA: Tricarboxylic acid
UPB1: Beta-ureidopropionase
UQCRH: Cytochrome b-c1 complex subunit 6
