# Supplementary figures and images for: Variable gene transcription underlies phenotypic convergence of hypoxia tolerance in sculpins
Source: BMC Evol Biol. 2018 Nov 3;18:163. doi: 10.1186/s12862-018-1275-1 (PMC6215679; doi:10.1186/s12862-018-1275-1)

## Smoothhead

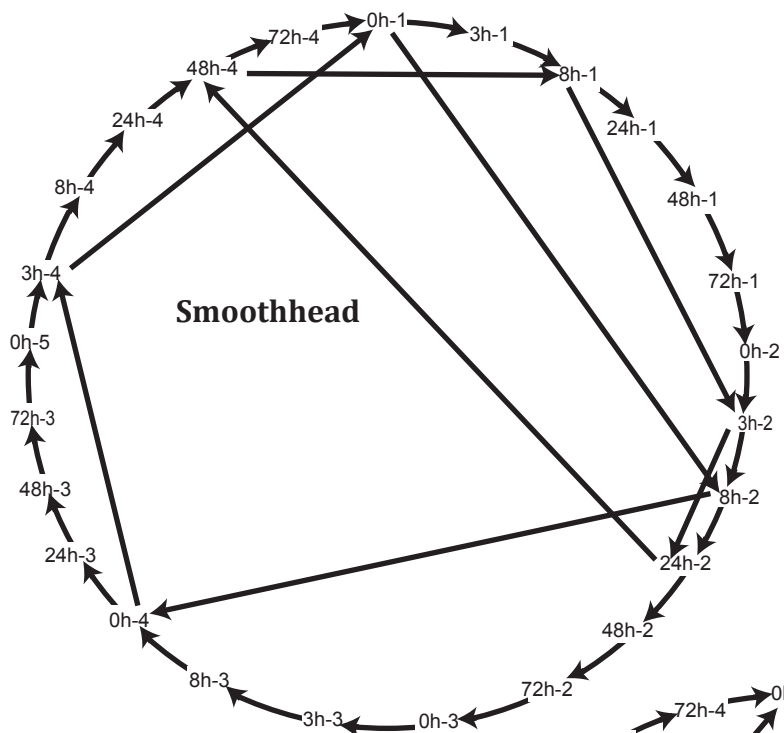

## Sailfin

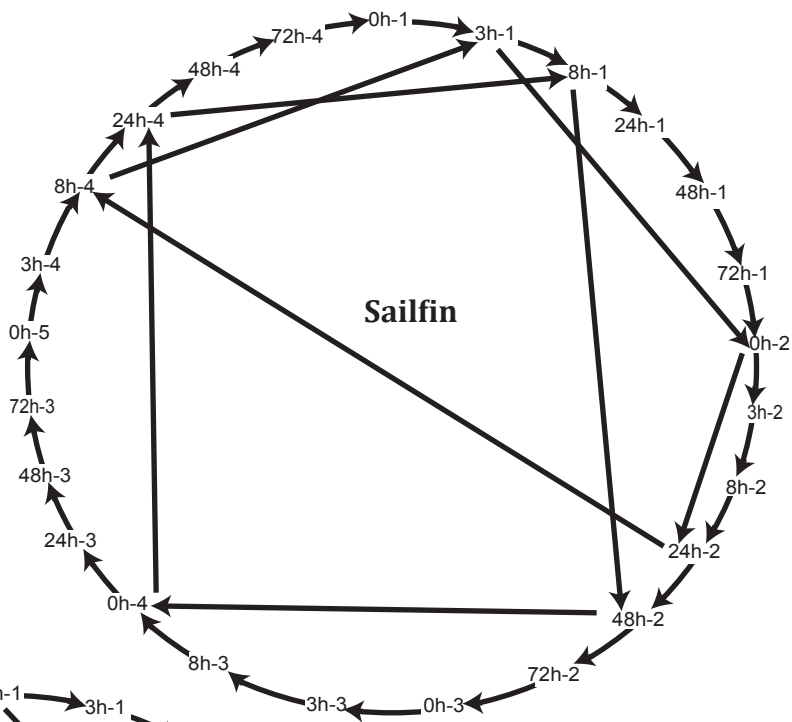

## Pacific Staghorn

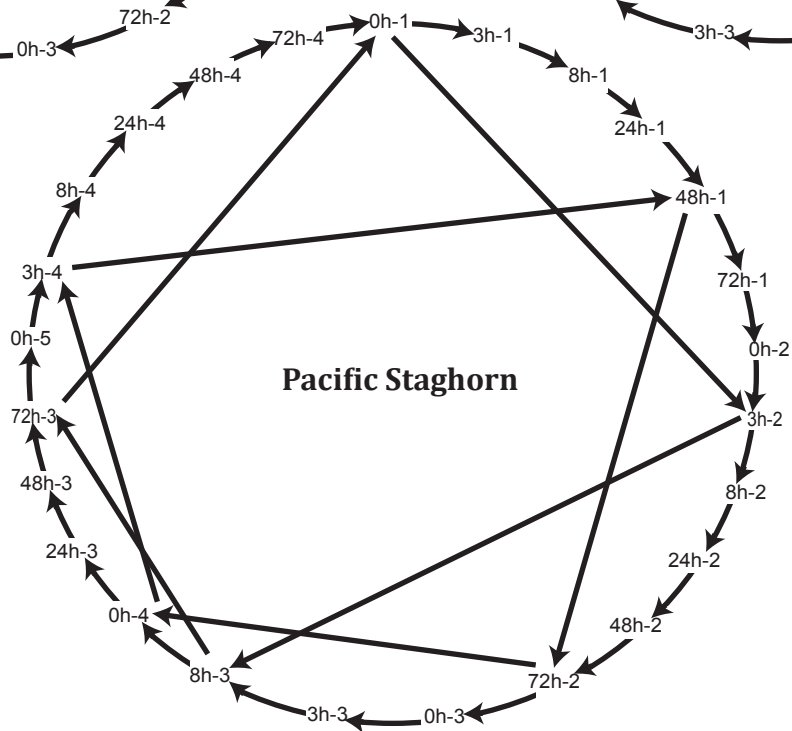

Supplement: Supplementary file 1 — Figure S1. Hybridization loop of 3 species of sculpin. Samples are identified first by the hours in hypoxia followed by the number of the biological replicate. (PDF 294 kb) [file 12862_2018_1275_MOESM1_ESM.pdf]

Log<sub>2</sub> Relative Expression

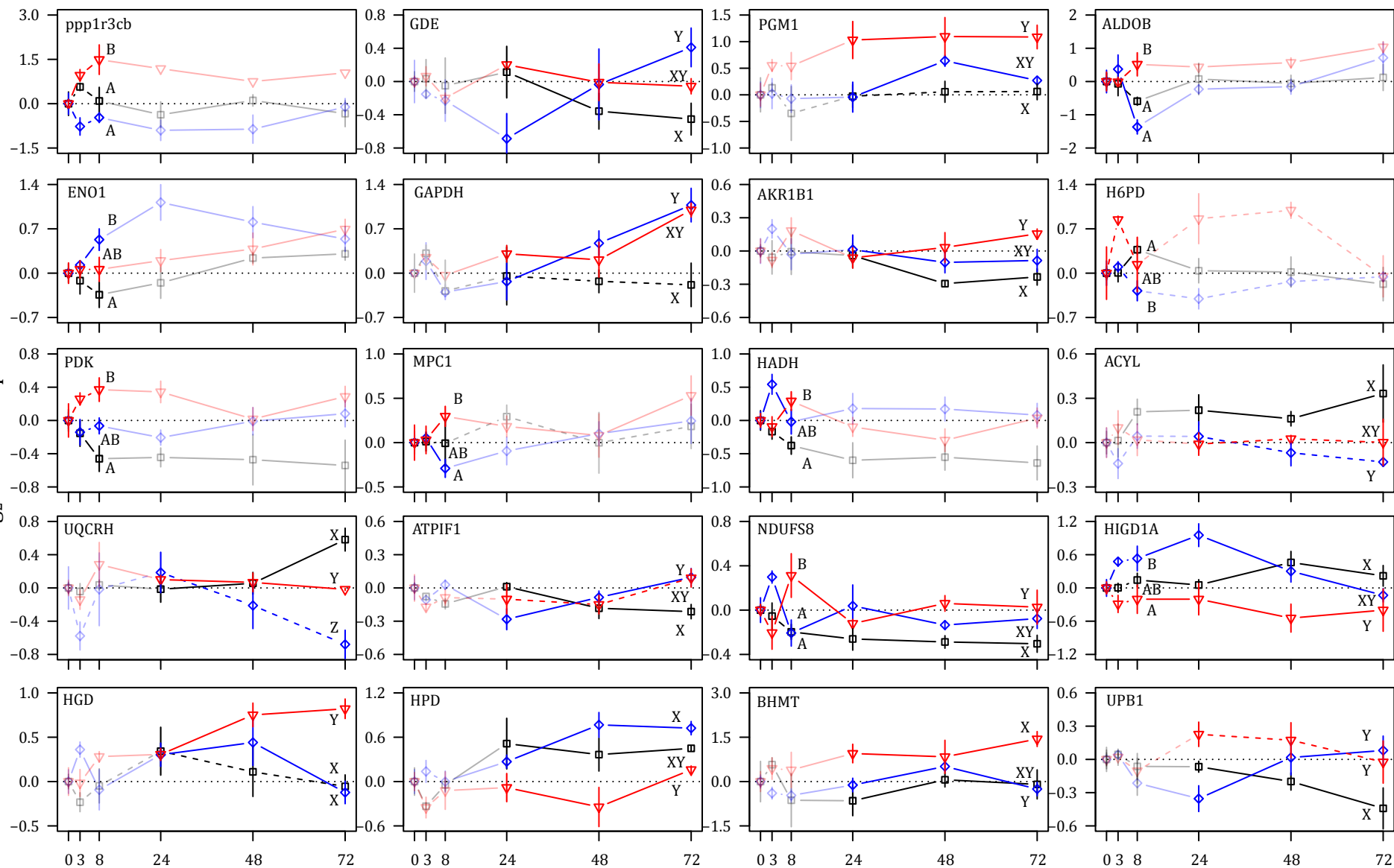

Time (hr)

Supplement: Supplementary file 2 — Figure S2. Transcript levels of genes associated with metabolism. Colours and symbols represent different species: the smoothhead sculpin (black line, square symbol), sailfin sculpin (blue line, diamond symbol) and Pacific staghorn sculpin (red line, inverted triangle symbol) exposed to 72 h of hypoxia. Solid lines represent the genes for which transcription significantly changed in response to hypoxia for a given species (q < 0.01) and dashed lines represent genes for which transcription did not significantly change in response to hypoxia. Opaque lines represent the portion of the time-course that is the focus of the difference among the species, while transparent lines represent the portion of the time-course for which transcription does not differ among the species. Letters represent significant difference in transcript levels between species (A-B represent difference in the short term hypoxia and X-Z represent differences in the long term hypoxia). Data are represented as mean ± SE. The genes in the panels are classified in the following categories: glycogen metabolic process (protein phosphatase 1 regulatory subunit 3C-B [ppp1r3cb], glycogen debranching enzyme [GDE] and phosphoglucomutase-1 [PGM1]), glycolysis (fructose-bisphosphate aldolase B [ALDOB], alpha-enolase [ENO1] and glyceraldehyde-3-phosphate dehydrogenase [GAPDH], polyol pathway (aldose reductase [AKR1B1]), the pentose-phosphate shunt (GDH/6PGL endoplasmic bifunctional protein [H6PD]), pyruvate metabolic process (pyruvate dehydrogenase kinase isozyme 4 [PDK] and mitochondrial pyruvate carrier 1 [MPC1]), fatty acid oxidation (hydroxyacyl-coenzyme A dehydrogenase [HADH]), tricarboxylic acid cycle (ATP-citrate synthase [ACYL]), oxidative phosphorylation (cytochrome b-c1 complex subunit 6 [UQCRH], ATPase inhibitor [ATPIF1], NADH dehydrogenase iron-sulfur protein 8 [NDUFS8] and HIG1 domain family member 1A [HIGD1A]), amino acid degradation (homogentisate 1,2-dioxygenase [HGD] and 4-hydroxyphenylpyruvate [file 12862_2018_1275_MOESM2_ESM.pdf]

Log<sub>2</sub> Relative Expression

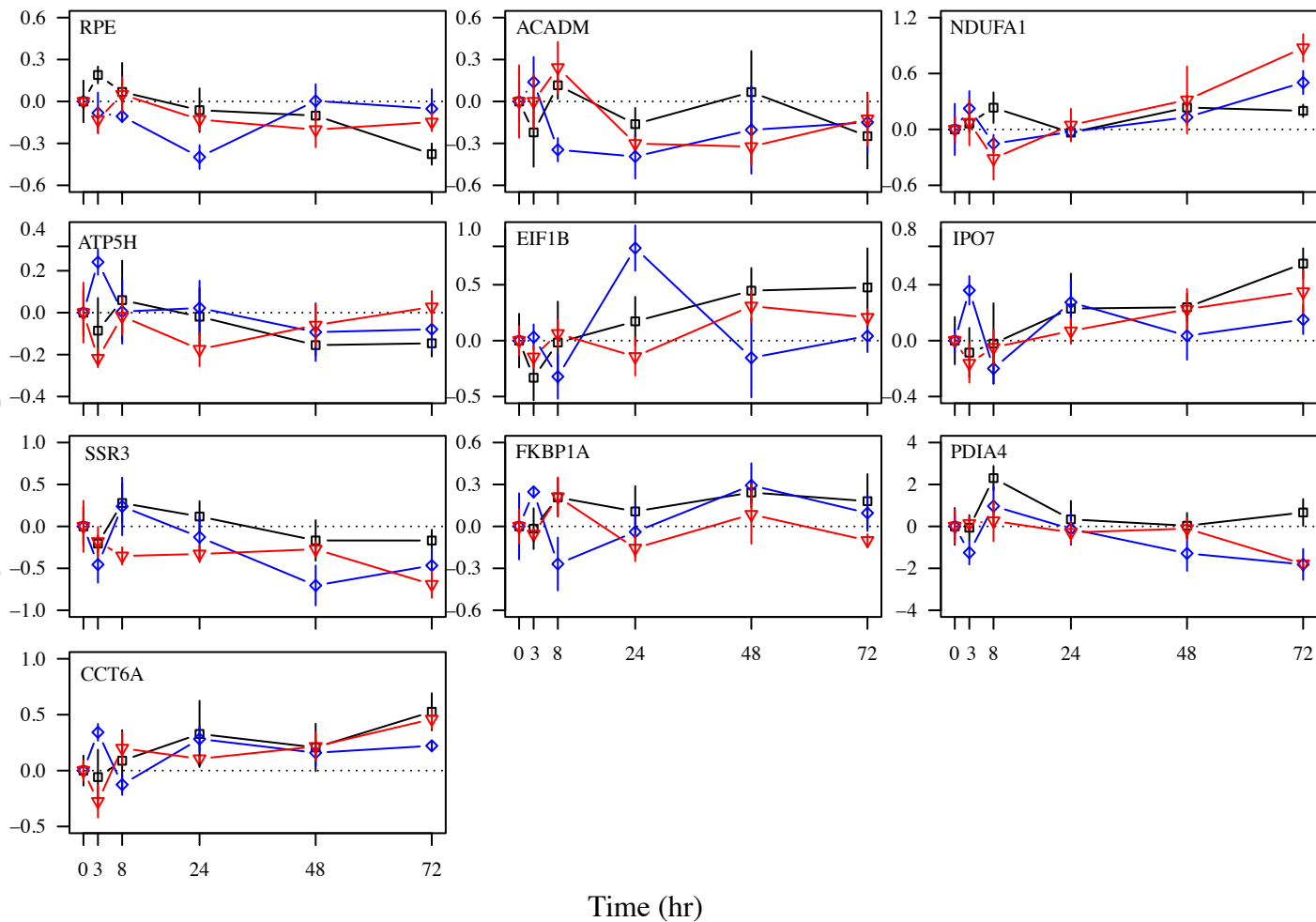

Supplement: Supplementary file 4 — Figure S4. Genes for which all three species showed similar significant changes in transcription in response to 72 h of hypoxia. The smoothhead sculpin is represented by the black line and square symbol, the sailfin sculpin is represented by the blue line and diamond symbol and the Pacific staghorn sculpin is represented by the red line and inverted triangle symbol. Data are represented as mean ± SE. The genes in the panels are classified in the following categories: pentose-phosphate shunt (ribulose-phosphate 3-epimerase [RPE]), fatty acid oxidation (medium-chain specific acyl-CoA dehydrogenase [ACADM]), oxidative phosphorylation (NADH dehydrogenase 1 alpha subcomplex subunit 1 [NDUFA1] and ATP synthase subunit d [ATP5H]), translational initiation (eukaryotic translation initiation factor 1b [EIF1B]), protein localization (importin-7 [IPO7] and translocon-associated protein subunit gamma [SSR3]) and protein folding (peptidyl-prolyl cis-trans isomerase FKBP1A [FKBP1A], protein disulfide-isomerase A4 [PDIA4] and T-complex protein 1 subunit zeta [CCT6A]). (PDF 136 kb) [file 12862_2018_1275_MOESM4_ESM.pdf]

Log<sub>2</sub> Relative Expression

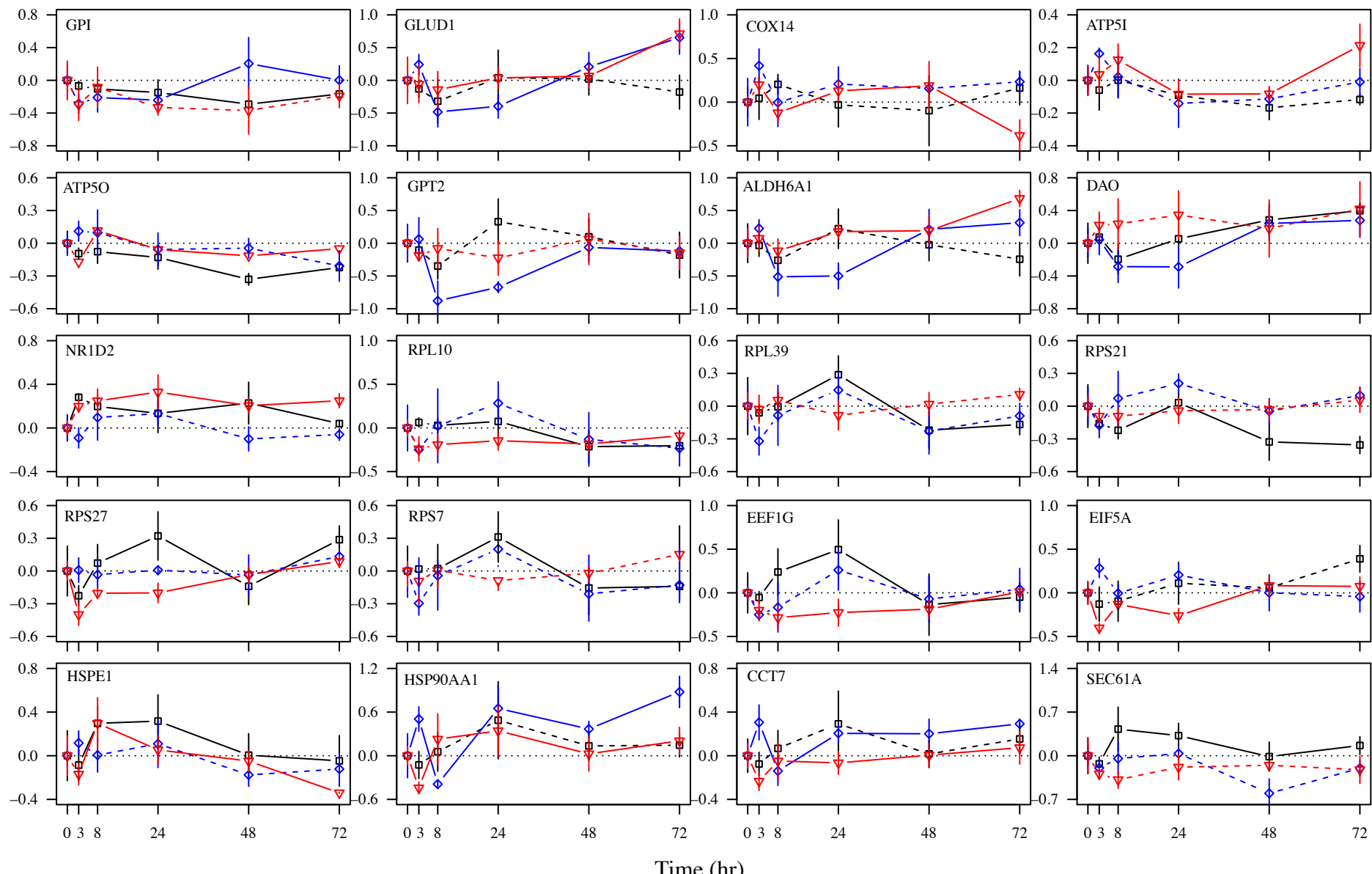

Supplement: Supplementary file 5 — Figure S5. Similar transcription patterns in genes for which only a subset of species showed a significant effect of time in hypoxia. The smoothhead sculpin is represented by the black line and square symbol, the sailfin sculpin is represented by the blue line and diamond symbol and the Pacific staghorn sculpin is represented by the red line and inverted triangle symbol. Solid lines represent the genes for which transcription significantly changed in response to hypoxia for a given species (q < 0.01) and dashed lines represent genes for which transcription did not significantly change in response to hypoxia. Data are represented as mean ± SE. The genes in the panels are classified in the following categories: glycolysis (glucose-6-phosphate isomerase [GPI]), tricarboxylic acid cycle (glutamate dehydrogenase [GLUD1]), oxidative phosphorylation (cytochrome c oxidase assembly protein COX14 [COX14], ATP synthase subunit e [ATP5I] and ATP synthase subunit O [ATP5O]), amino acid degradation (alanine aminotransferase 2 [GPT2], methylmalonate-semialdehyde dehydrogenase, acylating [ALDH6A1] and D-amino-acid oxidase [DAO]), transcriptional repression (nuclear receptor subfamily 1 group D member 2 [NR1D2]), ribosomal biogenesis (60S ribosomal protein L10 [RPL10], 60S ribosomal protein L39 [RPL39], 40S ribosomal protein S21 [RPS21], 40S ribosomal protein S27 [RPS27] and 40S ribosomal protein S7 [RPS7]), translational elongation (elongation factor 1-gamma [EEF1G] and eukaryotic translation initiation factor 5A-1 [EIF5A]), protein folding (10 kDa heat shock protein [HSPE1], heat shock protein HSP 90-alpha [HSP90AA1] and T-complex protein 1 subunit eta [CCT7]) and protein localization (protein transport protein Sec61 subunit alpha [SEC61A]). (PDF 194 kb) [file 12862_2018_1275_MOESM5_ESM.pdf]
