# Supplementary material for: Variable gene transcription underlies phenotypic convergence of hypoxia tolerance in sculpins
Source: BMC Evol Biol. 2018 Nov 3;18:163. doi: 10.1186/s12862-018-1275-1 (PMC6215679; doi:10.1186/s12862-018-1275-1)
Supplement: Supplementary file 3 — Figure S3. Transcript levels of genes associated with protein production, localization and folding. Colours and symbols represent different species: the smoothhead sculpin (black line, square symbol), sailfin sculpin (blue line, diamond symbol) and Pacific staghorn sculpin (red line, inverted triangle symbol) exposed to 72 h of hypoxia. The genes in the panels are classified in the following categories: transcriptional repression (histone deacetylase complex subunit SAP18 [SAP18] and carboxy-terminal domain RNA polymerase II polypeptide A small phosphatase 2 [CTDSP2]), RNA metabolic process (ribonuclease inhibitor [RNHI]), RNA processing (RNA-binding motif, single-stranded-interacting protein 2 [RBMS2] and ribosome biogenesis protein NSA2 homolog [NSA2] and 60S ribosomal protein L30 [RPL30]), translation (signal peptidase complex sunbunit 2 [SPCS2]), translational initiation (eukaryotic translation initiation factor 4E-binding protein 3 [EIF4EBP3], eukaryotic translation initiation factor 4E-1A [EIF4E1A] and eukaryotic translation initiation factor 4 gamma 2 [EIF4G2]), protein localization (ADP-ribosylation factor 5 [ARF5] and translocation protein SEC63 homolog [SEC63]), and protein folding (sulfhydryl oxidase 1 [QSOX1] and heat shock protein HSP 90-beta [HSP90AB1]). See Figure S1 legend for more detail. (PDF 346 kb) [file 12862_2018_1275_MOESM3_ESM.pdf]

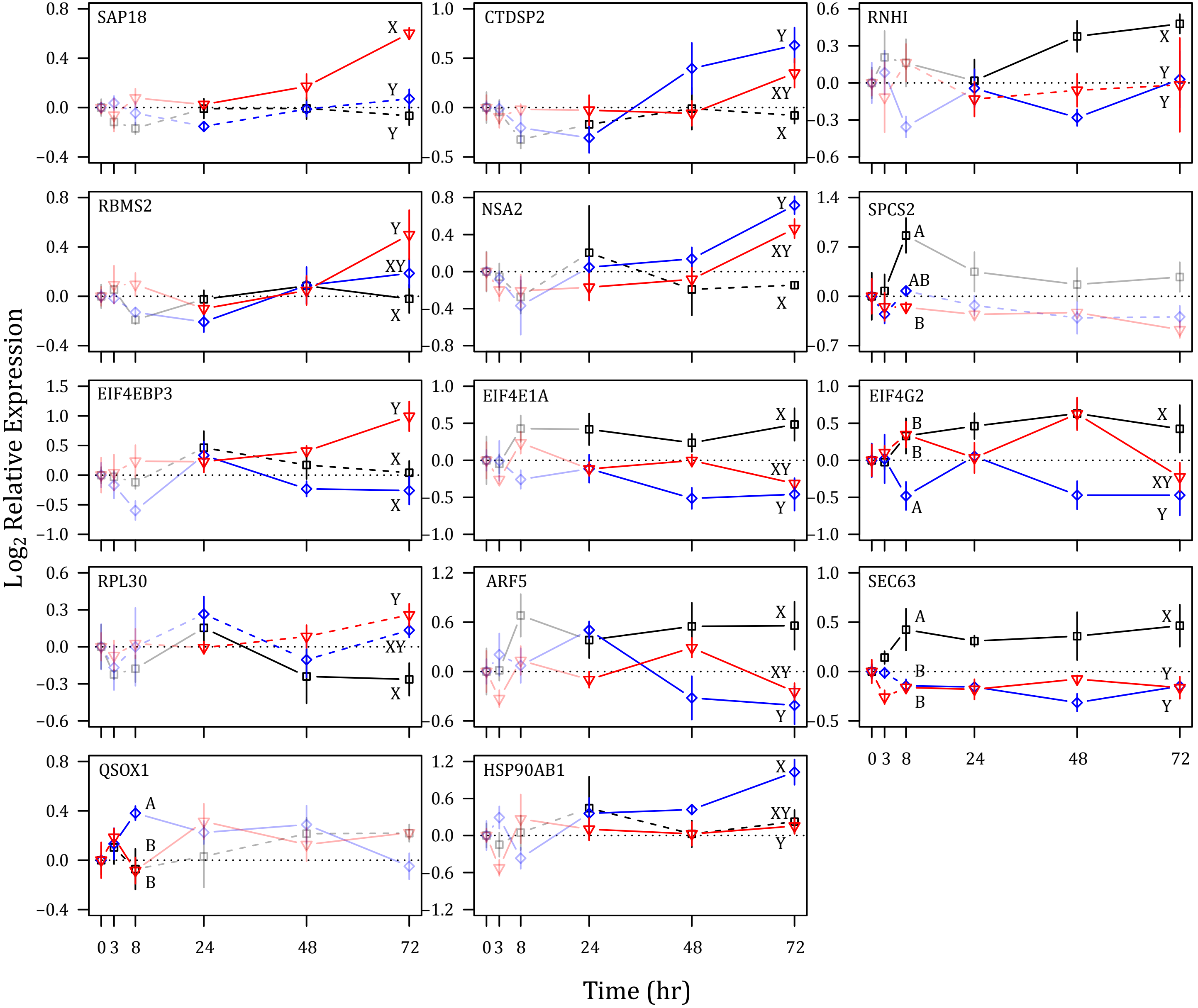

SAP18
CTDSP2
RNHI
RBMS2
NSA2
SPCS2
EIF4EBP3
EIF4E1A
EIF4G2
RPL30
ARF5
SEC63
QSOX1
HSP90AB1
Log$_2$ Relative Expression
Time (hr)
0 3 8 24 48 72
